# Joint trajectories of episodic memory and odor identification in older adults: patterns and predictors

**DOI:** 10.18632/aging.203280

**Published:** 2021-07-07

**Authors:** Christina S. Dintica, Miriam L. Haaksma, Jonas K. Olofsson, David A. Bennett, Weili Xu

**Affiliations:** 1 Aging Research Center, Department of Neurobiology, Care Sciences and Society, Karolinska Institutet, Stockholm, Sweden; 2 Department of Public Health and Primary Care, Leiden University Medical Center, Leiden, The Netherlands; 3 Gösta Ekman Laboratory, Department of Psychology, Stockholm University, Stockholm, Sweden; 4 Rush Alzheimer’s Disease Center, Rush University Medical Center, Chicago, IL 60612, USA; 5 Department of Epidemiology and Biostatistics, School of Public Health, Tianjin Medical University, China

**Keywords:** olfaction, memory, aging

## Abstract

Emerging evidence suggests that olfactory function is closely linked to memory function. The aims of this study were to assess whether olfactory and episodic memory functions follow similar age-related decline trajectories, to identify different patterns of decline, as well as predictors of the patterns.

1023 participants from the Memory and Aging Project were followed for up to 8 years with annual episodic memory and odor identification assessments. Trajectories were modelled using growth mixture models. Multivariate logistic regression was used to identify pattern predictors.

Three patterns of joint trajectories were identified; Class 1- stable average performance in both functions (*n*=690, 67.4%); Class 2- stable average episodic memory and declining odor identification (*n*=231, 22.6%); and Class 3- decline in both functions (*n*= 102, 10.0%). Class predictors included age, sex, *APOE* ε4 status, cognitive activity level and BMI. Participants in Class 3 were most likely to develop dementia.

Episodic memory and olfactory function show similar trajectories in aging. Such classification can contribute to a better understanding of the factors related to cognitive decline and dementia.

## INTRODUCTION

Olfactory function has been found to be closely related to episodic memory [1]. Structural and functional neuroimaging studies have shown that the olfactory nerve projects to several regions in the medial temporal lobe, [2] suggesting that the episodic quality of olfactory experiences might be due to this neuroanatomical overlap [3, 4].

Previous studies in older adults have shown that odor identification ability may predict decline in cognition, in particular episodic memory, as well as cognitive impairment and dementia [5–11]. Only a few studies thus far have examined the longitudinal relationship between olfaction and episodic memory [12–14]. One study reported that fluctuations in episodic memory correspond to fluctuations in olfaction over time, particularly for individuals with Alzheimer's disease (AD) pathology [12]. Moreover, the close overlap of olfactory and episodic memory areas in the middle temporal cortex, suggests that these two processes may decline together, possibly due to a shared relationship with neuropathological changes in mediotemporal lobe areas [15, 16].

Both odor identification and episodic memory decline with increasing age [17, 18]. Other factors, such as male sex, [19] apolipoprotein epsilon 4 (*APOE* ε4) status, [20] depression [21] and leisure activities [22–24] have been individually associated with odor or memory function. However, to our knowledge no study has addressed if episodic memory and olfactory abilities show joint vs distinctive age-related trajectories, and which factors may predict these patterns. Such classification would contribute to a better understanding of the factors related to cognitive decline and dementia.

The aims of this study were therefore, a) to examine the extent to which trajectories of episodic memory and odor identification decline are similar, b) to characterize patterns of episodic memory and odor identification trajectories, and c) identify the predictors of the observed patterns. To address these aims, we used data from a population-based study with annual follow-up examination over an 8-year interval.

## RESULTS

### Characteristics of the study population

The characteristics of the 1023 subjects are shown in Table 1. The majority, 794 (77.6%), were female, and the mean age at baseline was 78.2 (SD 7.5) years. The mean B-SIT and episodic memory (z-score) were 9.4 (SD 2.0) and 0.3 (SD 0.6) at baseline, respectively. During the follow-up period, among all participants, 290 (28.3%) participants died, and the participation rate of survivors exceeded 90%. The median number of assessments was 8 (IQR=8-5).

**Table 1:** Characteristics of the study sample (*n*= 1023) and per joint class membership.

**Characteristic**	**Values at baseline^a^**	**Class 1** **joint stable** ***n*= 690 (67.5%)**	**Class 2** **OI decline** ***n*= 231 (22.6%)**	**Class 3** **joint decline** ***n*=102 (10.0%)**	***P***
Women	794 (77.6)	550 (79.7)	157 (68.0)	87 (85.3)	<0.001
Age	79.4 (±7.4)	77.7 (±7.3)	82.5 (±6.1)^a^	83.8 (±5.8)^a^	<0.001
Years of education	15.1 (±3.2)	15.2 (±3.2)	14.9 (±3.2)	14.5 (±3.3)	0.113
Hypertension	553 (55.9)	379 (56.8)	120 (53.3)	54 (55.1)	0.652
Heart disease	79 (8.0)	46 (6.9)	25 (11.1)	8 (8.2)	0.130
Diabetes	90 (9.1)	61 (9.1)	21 (9.3)	8 (8.2)	0.946
BMI	27.7 (±5.4)	28.12 (±5.6)	27.03 (±4.7)^a^	25.9 (±5.0)^a^	<0.001
Depression	157 (15.8)	100 (14.9)	39 (17.3)	18 (18.4)^a^	0.535
Smoking status Never Current smoker	596 (58.3) 404 (39.5)	407 (59.0) 269 (39.0)	129 (55.8) 94 (40.7)	60 (59.4) 41 (40.6)	0.338
Previous smoker	22 (2.2)	14 (2.0)	8 (3.5)	0 (0.0)	
*APOE* ε4 carriers	213 (21.9)	134 (20.4)	46 (20.8)^a^	33 (33.7)^a^	0.011
Cognitive activity (hours per week)	3.2 (±0.7)	3.2 (±0.7)	3.2 (±0.7)	3.0 (±0.7)^a^	0.045
Social activity (hours per week)	2.7 (±0.6)	2.7 (±0.6)	2.6 (±0.6)^a^	2.6 (±0.6)^a^	<0.001
Physical activity (hours per week)	3.4 (±3.5)	3.6 (±3.7)	3.2 (±3.2)	3.1 (±3.1)	0.313
Episodic memory (z-score)	0.3 (±0.6)	0.4 (±0.5)	-0.1 (±0.7)^a^	-0.1 (±0.6)^a^	<0.001
B-SIT	9.4 (±2.0)	10.1 (±1.3)	7.8 (±2.2)^a^	7.8 (±2.4)^a^	<0.001
Nr of assessments		6.6 (±2.0)	6.3 (±2.1)	5.7 (±1.6)^a^	<0.001
Incident dementia		59 (8.6)	59 (25.5)^a^	69 (67.7)^a^	<0.001
Death during follow-up		144 (20.9)	84 (36.4)^a^	62 (60.8)^a^	<0.001

^a^Data are numbers (%) or means±(SD).

Abbreviations: *APOE* ε4, Apolipoprotein epsilon 4; B-SIT, Brief Smell Identification Test; BMI, body mass index.

### Heterogeneity of trajectories

**Figure 1:**
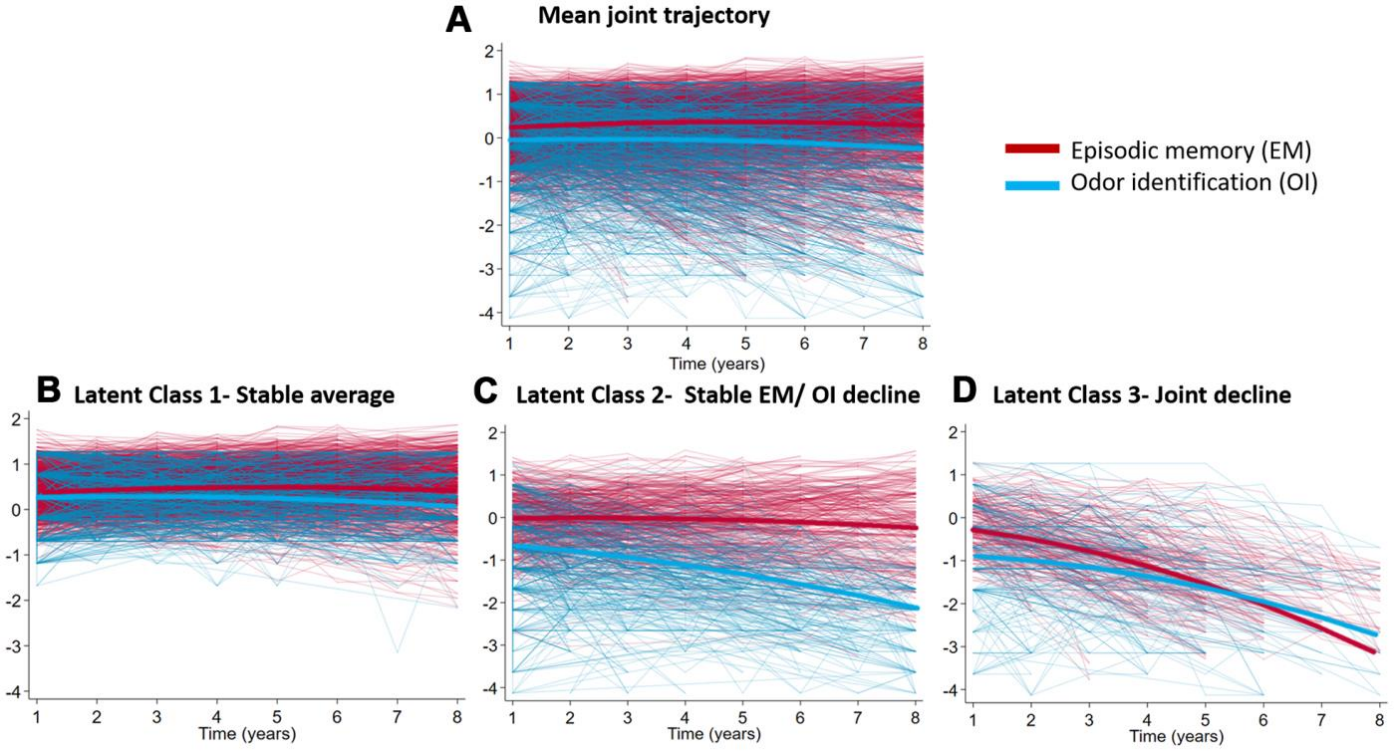
**Fitted and observed trajectories of jointly modelled episodic memory and B-SIT scores.** Scales are z-scores (mean 0, SD 1). (**A**) The trajectories of the entire sample (*n*= 1023; (**B**) Latent Class 1-Stable average (*n*= 690, 67.4%); (**C**) Latent Class 2- Stable EM/OI decline (*n*= 231, 22.6%); (**D**) Latent Class 3- Joint decline (*n*= 102, 10.0%). The mean trajectories of each plot are shown in bold.

Quadratic curves were fit for episodic memory and odor identification trajectories separately and in a joint model across 8 years. The observed individual trajectories of episodic memory and B-SIT means of the entire sample are depicted in Supplementary Figure 1A, 1E. The trajectories of B-SIT and episodic memory were clearly related, as shown by the positive correlation between their random slopes (1-class model; *r=* 0.57, *p* < .001). The joint trajectories mean of the entire sample is depicted in Figure 1A.

### Episodic memory

When fitting models with increasing numbers of classes, the 3-class model provided the best balance between model fit and model complexity, as confirmed by the LMR test (3- vs 4-class model: −2LL(4) = 1895.3, *p* = 0.321). An overview of the model fit criteria is shown in Supplementary Table 1. The difference in BIC between the 3-class model and the 4-class model was small, indicating that the model fit improvement caused by the 4^th^ class was minimal. The best-fitting 3-class model included a class-specific intercept variance and class-invariant slope variance. The parameter estimates of the 3-class model are shown in Supplementary Table 2, and the trajectories are depicted in Supplementary Figure 1B–1D. Class 1 was the largest class (*n=*799, 78.1%) and was characterized by above average episodic memory at baseline, and a stable trajectory over time. Class 2 was the second largest class (*n*=158, 15.4%), showing average episodic memory at baseline, as well as a quadratic decline over time. Class 3 was the smallest class (*n*=66, 6.5%), with below average episodic memory at baseline and faster decline over time.

### Odor identification

When we fitted models with increasing numbers of classes, the 3-class model provided the best fit according to the LMR test (3- vs 4-class model: −2LL(4) = 358.48, *p* = 0.095) and the class sizes (Supplementary Table 1). The difference in BIC between the 3-class model and the 4-class model was small, indicating that the model fit improvement caused by the 4^th^ class was minimal. The best-fitting 3-class model included a class-specific intercept variance and class-invariant slope variance. The parameter estimates of this 3-class model are shown in Supplementary Table 1 and the trajectories are depicted in Supplementary Figure 1F–1H. Class 1 was the largest (*n*= 731, 71.5%) and showed above average odor identification at baseline, which remained stable over time. Class 2 was the smallest class (*n*=79, 7.8%), showing around average odor identification performance at baseline, as well as a linear decline over time. Class 3 was the second largest (*n*=212, 20.7%), with the lowest odor identification at baseline, remaining rather stable over time.

### Episodic memory and odor identification

When we fitted models with increasing numbers of classes, the 3-class model provided the best balance between model fit and model complexity, as confirmed by the LMR test (3- vs 4-class model: −2LL(7)=908.95, *p*=0.345 (Supplementary Table 1). The difference in BIC between the 3-class model and the 4-class model was small, indicating that the model fit improvement caused by the 4^th^ class was minimal. The 3-class model with the best fit included a class-specific intercept variance and class-invariant slope variance. The parameter estimates of this 3-class model are shown in Table 2, and the trajectories are depicted in Figure 1B–1D. Participants in Class 1 (690, 67.4%) exhibited joint stable average performance, those in Class 2 showed stable average episodic memory and decline in odor identification (*n*=231, 22.6%), and Class 3 was characterized by joint decline in both episodic memory and odor identification (*n*=102, 10.0%).

**Table 2:** Parameter estimates for episodic memory and odor identification trajectories by latent class membership^a^.

**Parameters**	**Functions**	**Class 1- joint stable**	**Class 2 - OI decline**	**Class 3- joint decline**
		***n*=690 (67.4%)**	***n*=231 (22.6%)**	***n*= 102 (10.0%)**
		**mean (SE)**	**mean (SE)**	**mean (SE)**
**Fixed effects**				
Intercept	Episodic memory	0.441	0.008	-0.111
	Odor identification	0.370	-0.692	-0.855
Linear annual rate of decline	Episodic memory	0.067	0.013	-0.130
	Odor identification	0.035	-0.134	0.001
Quadratic annual rate of decline	Episodic memory	-0.008	-0.011	-0.036
	Odor identification	-0.009	-0.006	-0.043
**Random effects**				
Intercept variance	Episodic memory	0.130	0.346	0.322
	Odor identification	0.104	0.801	1.161
Linear slope variance	Episodic memory	0.004	0.004	0.004
	Odor identification	0.004	0.004	0.004
Residual variance baseline	Episodic memory	0.079	0.079	0.079
	Odor identification	0.384	0.384	0.384
Residual variance follow up 1	Episodic memory	0.09	0.09	0.09
	Odor identification	0.26	0.26	0.26
Residual variance follow up 2	Episodic memory	0.09	0.09	0.09
	Odor identification	0.33	0.33	0.33
Residual variance follow up 3	Episodic memory	0.09	0.09	0.09
	Odor identification	0.38	0.38	0.38
Residual variance follow up 4	Episodic memory	0.09	0.09	0.09
	Odor identification	0.41	0.41	0.41
Residual variance follow up 5	Episodic memory	0.08	0.08	0.08
	Odor identification	0.35	0.35	0.35
Residual variance follow up 6	Episodic memory	0.10	0.10	0.10
	Odor identification	0.45	0.45	0.45
Residual variance at follow up 7	Episodic memory	0.10	0.10	0.10
	Odor identification	0.39	0.39	0.39

^a^*n* was based on the final class counts of the estimated model. Note that individuals are in fact assigned a probability of class membership. OI, odor identification.

The sample characteristics of the three classes are shown in Table 1. Noteworthy is that 144 out of 690 (20.9%) participants died in Class 1 (stable average) during follow-up, 84 out of 231 (36.4%) died in Class 2 (average episodic memory/declining odor identification), and 62 out of 102 (60.8%) participants died in Class 3 (joint decline); 59 participants (8.6%) developed dementia over follow-up in Class 1, 59 (25.5%) in Class 2 and 69 (67.7%) in Class 3; participants in Class 1 were younger than those in Class 2 or 3, however the latter two classes did not differ significantly in age.

### Predictors of class membership

Having established 3 distinct patterns of decline and their relative occurrence, we next sought to determine what predictors could be used to characterize these groups. We first predicted decline outcomes for episodic memory, then for odor identification, and finally for both functions.

### Episodic memory

Using multivariable logistic regression, potential predictors of class membership listed in Table 1 were examined, with the class membership from the final 3-class model of episodic memory the dependent variable. This analysis was based on 914 participants; 109 (10.7%) were excluded due to missing values in covariates; the excluded participants did not differ significantly in B-SIT or episodic memory at baseline. Higher age at baseline and being an *APOE* ε4 carrier increased the likelihood of belonging to the lower performing Classes 2 and 3, relative to Class 1 (stable class). Higher BMI and cognitive activity decreased the likelihood of being in Class 3 relative to Class 1 (Supplementary Table 3).

### Odor identification

In the multivariable logistic regression, with the class membership from our final 3-class model of odor identification as the dependent variable, higher age at baseline was associated with higher odds of belonging to Class 2 relative to Class 1 (stable class). Moreover, higher baseline age and being an *APOE* ε4 carrier increased the likelihood of belonging to the lowest performing Class 3 relative to Class 1 (Supplementary Table 3).

### Episodic memory and odor identification

Table 3 shows the significant predictors of joint class membership presenting three patterns (joint stable, declining odor identification, and joint decline). Compared to the stable Class 1, people in the declining odor identification Class 2 were more likely to be older and male. People in the joint declining Class 3 were more likely to be older, *APOE* ε4 carriers, have a lower BMI, and be less engaged in cognitive activities (Table 3).

**Table 3:** Odds ratios (ORs) from multivariate prediction of joint class membership (*n* =914)^a^.

**Predictor**	**Class 2** **odor function decline** ***n*= 211**			**Class 3** **joint decline** ***n*= 94**	
	**OR (95% CI)**	***P* value**		**OR (95% CI)**	***P* value**
Age	1.11 (1.08 to 1.14)	<0.001		1.15 (1.10 to 1.20)	<0.001
Sex: male	1.98 (1.33 to 2.95)	0.001		0.73 (0.38 to 1.42)	0.350
Education	0.94 (0.89 to 1.00)	0.056		0.98 (0.90 to 1.07)	0.698
Smoking					
Previous	0.98 (0.62 to 1.39)	0.920		1.00 (0.61 to 1.63)	0.994
Current	2.11 (0.67 to 6.64)	0.200		0.44 (0.09 to 2.06)	0.299
*APOE* ε4 carrier	1.39 (0.92 to 2.12)	0.119		2.66 (1.57 to 4.52)	<0.001
Diabetes	1.02 (0.55 to 1.91)	0.941		1.43 (0.58 to 3.57)	0.437
Heart failure	1.23 (0.69 to 2.20)	0.488		1.08 (0.44 to 2.70)	0.861
Hypertension	0.77 (0.54 to 1.08)	0.132		0.79 (0.48 to 1.29)	0.343
BMI	0.98 (0.95 to 1.02)	0.309		0.93 (0.88 to 0.98)	0.007
Depression	1.50 (0.95 to 2.38)	0.082		1.30 (0.68 to 2.50)	0.423
Social activity (hours per week)	0.83 (0.61 to 1.13)	0.236		0.82 (0.53 to 1.25)	0.348
Cognitive activity (hours per week)	1.00 (0.75 to 1.32)	0.981		0.62 (0.43 to 0.89)	0.010
Physical activity (hours per week)	0.99 (0.94 to 1.04)	0.661		0.98 (0.91 to 1.06)	0.609

Reference: Class 1-Joint stable (*n*=609).

Abbreviations: *APOE* ε4, apolipoprotein epsilon 4; BMI, body mass index.

^a^N= 109 missing information on any 1 or more predictors.

### Sensitivity analysis

As both decline in episodic memory and olfaction are strongly related to aging, we conducted a sensitivity analysis by rerunning the models of joint prediction of class membership for episodic memory and odor identification, adjusting for age at baseline. The best-fitting 3-class model included a class-specific intercept variance and class-invariant slope variance.

The parameter estimates of the 3-class model are depicted in Supplementary Table 4. Participants in Class 1 (747, 73.0%) exhibited joint stable average performance, those in Class 2 showed stable average episodic memory and decline in odor identification (*n*=216, 21.1%), and Class 3 was characterized by joint decline in both episodic memory and odor identification (*n*=60, 6.0%).

## DISCUSSION

In this study of community-dwelling older adults with long-term follow-up, we found that episodic memory and odor identification show similar aging trajectories. We identified three distinct patterns, using 8 years of longitudinal data on episodic memory and odor identification. These patterns were characterized by 1) average performance in both episodic memory and odor identification stable over time; 2) decline in odor identification only; and 3) joint decline in both functions. The patterns were associated with variables such as age, sex, *APOE* ε4 carrier status, BMI, cognitive activity, and were differently related to key outcomes such as dementia and death.

Previous research suggests that odor identification impairment may reflect an accumulation in brain pathology affecting brain areas involved in both olfaction and episodic memory [25–27]. Studies from MAP have previously shown that impaired baseline odor identification is associated with a steeper decline in episodic memory and smaller volumes in the hippocampus and entorhinal cortex [10, 28]. Individuals with low odor identification performance and high AD pathology have faster episodic memory decline, [12] and post-mortem AD pathology in mesiotemporal brain regions may account for 12% of the variation in odor identification before death [27]. Evidence from other cohort studies suggests that odor identification may serve as an early marker of incipient cognitive impairment and dementia [5–11]. To the best of our knowledge, this is the first study categorizing functional decline in terms of joint trajectories of episodic memory and odor identification.

We found that odor identification and episodic memory were correlated over time, showing similar trajectories. Our findings are compatible with those of a recent study, which showed that episodic memory scores and B-SIT scores vary together over time using B-SIT scores as a time-varying covariate when modelling episodic memory decline [12]. The authors of the previous study also reported that there was a stronger association between B-SIT and episodic memory variation in persons with intermediate to high AD pathology. The evidence thus points to AD pathology as a mediator between the link between decline in both behavioral domains. Indeed, the present study found that being an *APOE* ε4 carrier, a known risk factor for AD, [29] increased the likelihood of belonging to the “concurrent decline” class by almost 3-fold. A previous study found that episodic memory decline was associated with odor identification impairment only in ɛ4-carriers, suggesting that the ɛ4 is involved in the functional association between ongoing episodic memory decline and olfaction [30]. In a study investigating “change-change” correlation in episodic memory and odor identification, the correlation was only seen in *APOE* ε4 carries, specifically ε4 homozygotes [13]. This is consistent with evidence showing that ɛ4-carriers with Alzheimer’s dementia, compared to those with no ɛ4 allele, exhibit cortical atrophy patterns more pronounced in mesialtemporal lobe regions, supporting olfactory and episodic memory functions [31].

In two out of the three joint classes identified (stable Class 1 and declining Class 3), odor identification and episodic memory progressed in parallel. This is in line with evidence showing a close overlap of olfaction and episodic memory areas in the middle temporal cortex, and would suggest that these two processes reflect the integrity of mesiotemporal lobe areas, possibly due to a shared relationship with pathological changes linked to neurofibrillary tangles [15, 16]. Interestingly, in Class 2, episodic memory had a stable average trajectory, whereas odor identification was steadily declining. This may seem contrary to the patterns observed in the other two classes. However, compared to memory structures, the olfactory system has been shown to be especially sensitive to non-dementia related pathology, and environmental agents [32]. Olfactory decline is quite common in old age, and such decline may be caused by a wide variety of factors, including accumulated environmental damage to the olfactory epithelium, changes in nasal airflow and mucus composition, or declining sensitivity and tuning of receptor neurons, all of which would contribute to poor or declining olfactory performance [33, 34]. We speculate that Class 2, characterized by declining odor identification but retained memory ability, is to a large extent made up of individuals with peripheral olfactory dysfunction.

In addition to the *APOE* ε4 carrier status, we found that age, sex, BMI, and cognitive activity were predictors of joint class membership. The findings are consistent with previous studies showing that both odor identification and episodic memory decline with increasing age [18, 35]. Male sex has been associated with lower olfactory performance in previous studies, [19] and was also a predictor of Class 2 membership in the current study. Higher BMI was associated with lower probability of belonging to Class 3, which is in line with an emerging body of work reporting high BMI appearing to be protective of dementia due to weight loss in pre-clinical stages, [36] as this class had the highest number of individuals who were diagnosed with dementia over follow-up. Lastly, a low engagement in cognitive activities predicted joint class membership. Such activities are postulated to be protective against dementia and to delay the clinical manifestation of AD pathology as “resilience” factors [23, 24, 37]. In the joint declining class, cognitive activity at baseline was reduced, which may partly explain the steep joint decline. However, whether or not the decline in cognitive activity contributed to cognitive decline or may be a reflection of it, cannot be determined in this study. In addition, Class 3 had the highest number of incident dementia cases as well as deaths over the follow-up, suggesting that a steep decline in episodic memory as well as odor identification may be indicative of a particularly vulnerable group with rapid deterioration in cognition and higher mortality.

The strengths of this study include the annual follow-up assessments of episodic memory and odor identification, the composite score of episodic memory created from several tests, the coordinated univariate analysis of odor identification and episodic memory which characterized trajectories and their predictors separately, and the joint modelling, allowing for the characterization of these processes together and their shared predictors. Moreover, the B-SIT has been found to be valid in most cross-cultural settings, making it possible to generalize our findings to other cultures than North America [17]. The limitations of this study are that we relied on only one test for odor identification which made this measure more susceptible to missing values as well as ceiling and floor effects. Further, the measure cannot separate different aspects of olfactory dysfunction (e.g. detection, quality discrimination or word-odor matching). Moreover, the MAP study participants were recruited from retirement facilities and were on average highly educated, which may limit the generalizability to other populations. Related to this issue, the study sample was restricted to those with at least one concurrent assessment of episodic memory and odor identification. The excluded participants were older, were in worse physical health and less educated, which likely would have implications for the trajectories we identified biasing towards an underestimation of decline, especially as two of the classes had little to no decline in episodic memory during the eight years of follow up. In addition, the GMMs handle missing data patterns assuming missing at random, this assumption may not hold, with the implication of potentially underestimating the trajectories of decline in episodic memory and odor identification, due to selective survival. Furthermore, odor identification and episodic memory were tested annually, thus there may be practice effects, which may underestimate decline. However, in previous studies in MAP, we have seen little evidence that practice would substantially diminish individual differences in rates of change [10, 38]. Lastly, we did not have a comprehensive comorbidity assessment available to include as a predictor of class membership; comorbidities are common in old age and may contribute to both worse episodic memory [39] and odor identification [19].

In conclusion, this study points to the interrelation between episodic memory and olfaction in old age, which may reflect their shared vulnerability to changes in the medio-temporal lobes.

## MATERIALS AND METHODS

### Study population

The Rush Memory and Aging Project (MAP) is an ongoing prospective clinical-pathologic study on risk factors for common chronic conditions of old age. Eligibility for participation involves agreeing to annual clinical evaluations and to the donation of brain, spinal cord, and selected nerves and muscles to Rush investigators at death. Detailed information on the MAP study design and the evaluation protocol is provided elsewhere [39]. In brief, participants were recruited from church groups, senior centers, retirement communities, and senior citizen housing facilities, within the greater Chicago area. At baseline and thereafter, all participants underwent extensive clinical assessments, including detailed medical history, neurological examination, extensive cognitive function testing, and odor identification testing.

Enrollment began in 1997. However, annual olfaction testing did not start until 2011, therefore 2011 serves as the analytic baseline for these analyses. Through 2018, 1041 participants were enrolled and annually followed-up. Out of the 1041 participants, we excluded participants with prevalent dementia (*n* = 18), leaving 1023 for the current analyses.

The study was approved by the Institutional Review Board of Rush University Medical Center and was performed in accordance with the ethical standards laid down in the 1964 Declaration of Helsinki and its later amendments. Informed consent was obtained from all participants. All participants signed an Anatomic Gift Act for organ donation. Participants also signed a repository consent that allowed their data to be shared. More information on obtaining data can be found on the RADC Resource Sharing Hub at https://www.radc.rush.edu.

### Data collection

Participants underwent uniform evaluations with trained staff including structured interviews, clinical and neurological examinations, and cognitive testing as described previously [39]. Data on socio-demographic characteristics (i.e., age, sex, and education), lifestyle factors (i.e., smoking), medical conditions, and cognitive function were collected at each wave following standardized procedures [39].

Education was recorded as participants’ maximum years of formal schooling. Smoking was categorized as “never smoked”, “former smoker” and “current smoker”. Self-reported information on medical conditions including heart disease, hypertension, diabetes was collected during the interview at baseline [39]. at study entry blood samples were taken and the *APOE* gene was genotyped utilizing high-throughput sequencing. Participants were stratified as epsilon 4 (ε4) carriers or ε4 non–carriers. Depression was determined according to the criteria of the Diagnostic and Statistical Manual of Mental Disorders, 3rd ed, Revised, implemented with a subset of questions from the Diagnostic Interview Schedule at baseline [40]. Clinical diagnosis of dementia was conducted on the basis of criteria of the joint working group of the National Institute of Neurological and Communicative Disorders and Stroke and the Alzheimer’s disease and Related Disorders Association (NINCDS/ADRDA) [41].

### Outcomes

### Odor identification

At the analytic baseline and at each follow-up, the Brief Smell Identification Test (B-SIT) (Sensonics. Inc., Haddon Heights, USA) was administered. The B-SIT is a 12-item standardized test, with 4 alternatives per item. In this test, a booklet is presented containing a scratchable patch of microencapsulated odorant on each page. The examiner scratches the odor patch with a pencil to release the odorant and places it under the participant’s nose, the participant is then asked which of four specific odors the sample most closely resembles. The score reflects the number of odors correctly recognized, with possible scores ranging from 0-12. If an item response is missing for a maximum of two, a score of 0.25 is assigned, corresponding to a chance level performance. If responses to three or more items are missing, data on this test are considered missing. The B-SIT has previously shown to be internally consistent, with good correspondence to the 40-item University of Pennsylvania Smell Identification Test from which it was derived [27].

### Episodic memory

Episodic memory was measured using CERAD (Consortium to Establish a Registry for Alzheimer’s Disease) Word List Memory, Recall, and Recognition, immediate and delayed recall of the East Boston Story, and Logical Memory immediate and delayed recall. Raw scores on each test were converted to z-scores (i.e. standardized based on all MAP participants at baseline) and then the average z-score among the tests for episodic memory was computed, as reported in detail in a previous study [28].

### Assessment of lifestyle factors

Three measures of stimulating mental, social and physical activities were recorded during the baseline interview.

### Cognitive activities

Participants completed a 7-item cognitive activity questionnaire [22, 36]. Activities include reading, writing letters, visiting a library, and playing games such as chess or checkers. Frequency of participation in each activity was rated from 1 (once a year or less) to 5 (every day or about every day) [36].

### Social activity

The frequency of activity was assessed on a 6-item scale including: a) going to restaurants or sporting events; b) going on day or overnight trips; c) doing unpaid community or volunteer work; d) visiting relatives’ or friends’ houses; e) participating in groups, such as a senior center; and f) attending church or religious services. Participants were asked to rate how often they participated in each activity based on a 5-point scale from 1 (once a year or less) to 5 (every day or about every day) [36].

### Physical activity

Frequency of activity was recorded as the hours per week participants reported engagement in 5 categories of activities: walking for exercise, gardening or yard work, calisthenics or general exercise, bicycle riding (including stationary bikes), and swimming or water exercises [36].

### Predictors on class membership

The following variables were included as potential predictors of class membership: age (years); sex (male/female); education (years); history of hypertension (yes/no), depression (yes/no), smoking (never/previous/current,) *APOE* ε4 status (any ε4/no ε4), diabetes (yes/no), history of heart failure (yes/no), BMI (continuous), social activity (hours per week), cognitive activity (hours per week), physical activity (hours per week).

### Statistical analyses

We used growth mixture models (GMMs) to model trajectories of episodic memory and odor identification separately and jointly over time. We used yearly assessments of episodic memory and odor identification to model the trajectories. The time scale was time from the first assessment (2011) until last follow-up (2018), maximum 8 years. Episodic memory and odor identification were assessed yearly; thus the timescale is in years. The GMM is a longitudinal form of latent class analysis, using mixed models. The GMMs therefore groups participants into latent classes, on the basis of similarities in their trajectory patterns over time. This is done by fitting an increasing number of curves until an optimal balance between model fit and model complexity is reached. Quadratic models with 1 to 5 classes were fit, and the final model was chosen based on the Bayesian information criterion (BIC), Lo-Mendell-Rubin (LMR) likelihood ratio test, and class size. The BIC indicates the fit of a model, the lower the value, the better the fit of the model is. The LMR test is used to compare model fit between 2 nested models. A significant LMR test indicates that the model with *k* classes has a better fit than the same model with *k − 1* classes. The parameter estimates were obtained using maximum likelihood estimation, with standard errors (SEs) that are robust to non-normality. The quadratic slope variance was fixed to zero. For ease of interpretation and presentation, the B-SIT scores were standardized (based on all participants at baseline) for comparability with the global cognition z-score. For parsimony, the residual variances assumed to be equal across classes and were allowed to vary over time. After the model with the best fit was decided, we examined which factors predicted class membership using multinomial logistic regression with the 3-step method in a multivariable model [42]. The GMMs were fit using Mplus version 8.2. Further analyses and processing of results and multinomial logistic regression models, were performed using Stata v. 15 and RStudio v. 1.2.5001.

## Supplementary Material

Supplementary Figure 1

Supplementary Tables
