# Supplementary material for: Joint trajectories of episodic memory and odor identification in older adults: patterns and predictors
Source: Aging (Albany NY). 2021 Jul 7;13(13):17080–96. doi: 10.18632/aging.203280 (PMC8312450; doi:10.18632/aging.203280)
Supplement: Supplementary Figure 1 [file aging-13-203280-s001.pdf]

## SUPPLEMENTARY FIGURE

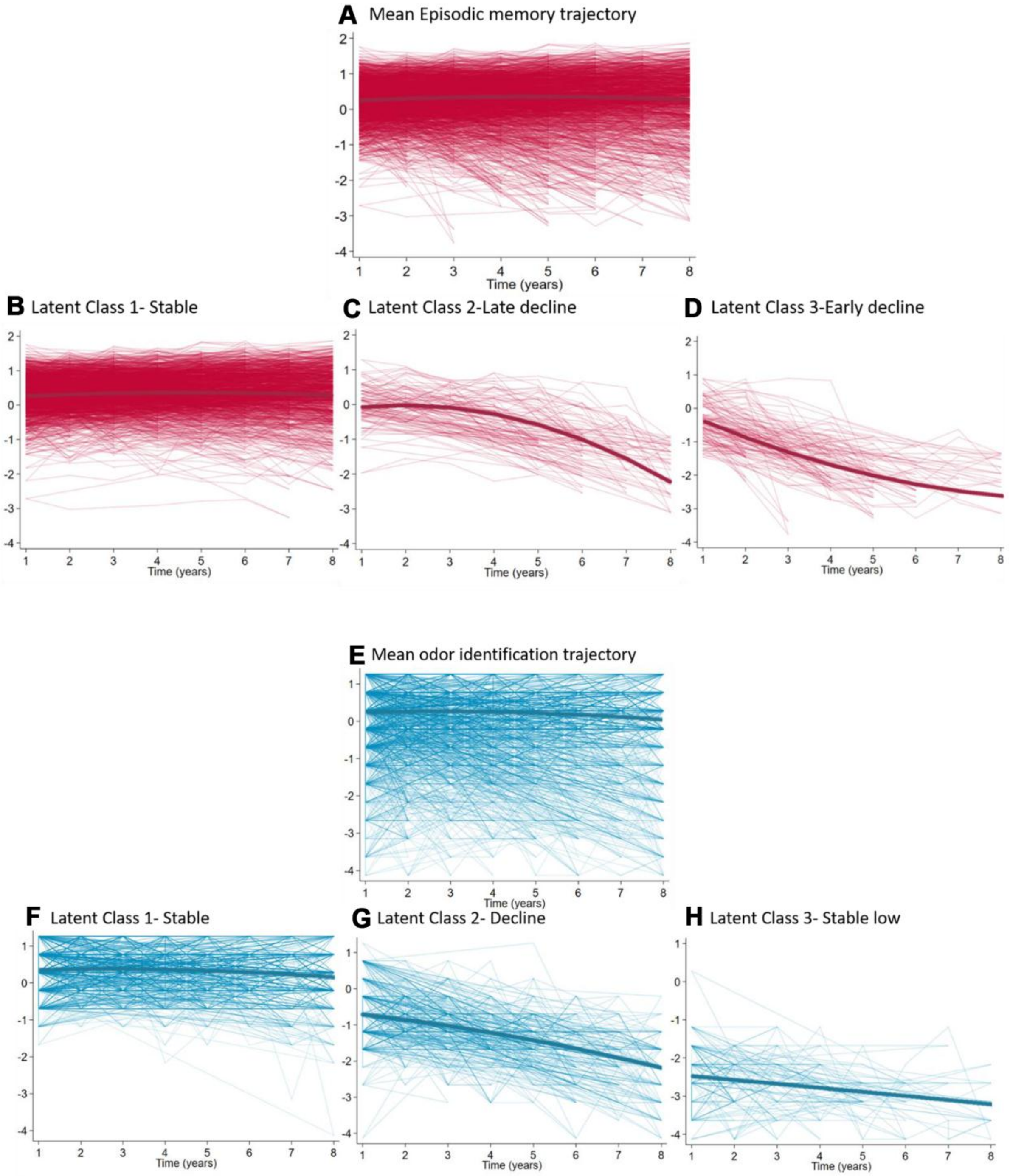


**Supplementary Figure 1. Fitted and observed trajectories of episodic memory and B-SIT scores (*n*= 1023).** Scales are z-scores (mean 0, SD 1). (**A**) Sample episodic memory trajectory; (**B**) Latent episodic memory Class 1- Stable average (*n* = 799, 78.1%); (**C**) Latent episodic memory Class 2- Late decline (*n* = 158, 15.4%); (**D**) Latent episodic memory Class 3-Early decline (*n* = 66, 6.5%); (**E**) Sample mean odor identification trajectory; (**F**) Latent odor identification Class 1-Stable average (*n*=731, 71.5%); (**G**) Latent odor identification Class 2-Decline (*n*= 79, 7.8%); (**H**) Latent odor identification Class 3-Stable low (*n*=213, 20.8%). The mean trajectories of each plot are shown in bold.
