# Supplementary material for: Joint trajectories of episodic memory and odor identification in older adults: patterns and predictors
Source: Aging (Albany NY). 2021 Jul 7;13(13):17080–96. doi: 10.18632/aging.203280 (PMC8312450; doi:10.18632/aging.203280)
Supplement: Supplementary Tables [file aging-13-203280-s002.pdf]

SUPPLEMENTARY TABLES

Supplementary Table 1. Overview of class enumeration.

Episodic memory					
**No. classes**	**No. parameters**	**BIC**	**Entropy**	**LMR *P* value**	**Smallest class size, %**
1	11	14578.62	-	-	-
2	15	11150.20	0.922	<0.001	22.9
3	19	9312.36	0.890	0.035	6.5
4	23	8461.28	0.635	0.321	9.2
**Odor identification**					
**No. classes**	**No. parameters**	**BIC**	**Entropy**	**LMR *P* value**	**Smallest class size, %**
1	11	11172.82	-	-	-
2	15	9469.73	0.872	<0.001	22.9
3	19	9025.78	0.827	0.039	7.8
4	23	8855.41	0.758	0.095	6.0
**Episodic memory and odor identification**					
**No. classes**	**No. parameters**	**BIC**	**Entropy**	**LMR *P* value**	**Smallest class size, %**
1	22	25751.45	-	-	-
2	29	21473.49	0.941	<0.001	22.6
3	36	19617.32	0.894	<0.001	10.0
4	43	18756.89	0.757	0.345	8.4

Supplementary Table 2. Parameter estimates for episodic memory and odor identification trajectories by latent class.

	Episodic memory			Odor identification		
	**Class 1**	**Class 2**	**Class 3**	**Class 1**	**Class 2**	**Class 3**
Prevalence ([*n*][a], %)	799 (78.1)	158 (15.4)	66 (6.5)	731 (71.5)	79 (7.8)	213 (20.8)
**Fixed effects**	Mean (SE)	Mean (SE)	Mean (SE)	Mean (SE)	Mean (SE)	Mean (SE)
Intercept	0.398	-0.090	-0.135	0.384	-0.082	-1.079
Linear annual rate of decline	0.007	0.005	-0.430	0.024	-0.579	0.107
Quadratic annual rate of decline	-0.007	-0.023	0.011	-0.007	0.036	-0.039
**Random effects**						
Intercept variance	0.182	0.352	0.336	0.053	0.464	1.033
Linear slope variance	0.001	0.001	0.001	0.001	0.001	0.001
Residual variance at baseline	0.085	0.085	0.085	0.338	0.338	0.338
Residual variance at follow up 1	0.091	0.091	0.091	0.262	0.262	0.262
Residual variance at follow up 2	0.088	0.088	0.088	0.324	0.324	0.324
Residual variance at follow up 3	0.087	0.087	0.087	0.370	0.370	0.370
Residual variance at follow up 4	0.091	0.091	0.091	0.418	0.418	0.418
Residual variance at follow up 5	0.087	0.087	0.087	0.335	0.335	0.335
Residual variance at follow up 5	0.119	0.119	0.119	0.463	0.463	0.463
Residual variance at follow up 7	0.137	0.137	0.137	0.453	0.453	0.453

[a]*n* was based on the final class counts of the estimated model. Note that individuals are in fact assigned a probability of class membership.

Supplementary Table 3. Odds ratios (ORs) from multivariate prediction of class membership (*n* = 914)[a].

Episodic memory	Class 2 *n*= 148		Class 3 *n*= 58	
	**OR (95% CI)**	***P* value**	**OR (95% CI)**	***P* value**
Age	1.13 (1.09 to 1.16)	<0.001	1.11 (1.06 to 1.16)	<0.001
Sex: male	1.40 (0.88 to 2.21)	0.153	0.44 (0.17 to 1.10)	0.079
Education	0.98 (0.92 to 1.05)	0.603	0.98 (0.88 to 1.08)	0.675
Smoking				
Previous	1.09 (0.73 to 1.62)	0.674	0.79 (0.44 to 1.45)	0.451
Current	2.00 (0.50 to 8.07)	0.330	0.73 (0.23 to 2.36)	0.598
APOE ε4 carrier	2.17 (1.40 to 3.38)	0.001	2.80 (1.49 to 5.27)	0.001
Diabetes	0.49 (0.20 to 1.20)	0.118	1.33 (0.47 to 3.80)	0.595
Heart failure	0.71 (0.35 to 1.47)	0.357	1.18 (0.41 to 3.37)	0.759
Hypertension	0.89 (0.60 to 1.33)	0.579	1.09 (0.60 to 1.99)	0.771
BMI	0.98 (0.94 to 1.02)	0.240	0.93 (0.87 to 0.99)	0.023
Depression	1.40 (0.83 to 2.36)	0.209	1.50 (0.73 to 3.09)	0.270
Social activity (hours per week)	0.78 (0.55 to 1.11)	0.165	0.66 (0.39 to 1.11)	0.117
Cognitive activity (hours per week)	0.77 (0.56 to 1.05)	0.101	0.48 (0.31 to 0.74)	0.001
Physical activity (hours per week)	1.083(0.97 to 1.09)	0.304	0.93 (0.83 to 1.04)	0.221
**Odor identification**	**Class 2 *n*= 74**		**Class 3 *n*=194**	
	**OR (95% CI)**	***P* value**	**OR (95% CI)**	***P* value**
Age	1.11 (1.08 to 1.14)	<0.001	1.11 (1.08 to 1.15)	<0.001
Sex: male	1.60 (0.89 to 2.90)	0.115	1.29 (0.84 to 1.96)	0.243
Education	0.98 (0.90 to 1.07)	0.653	0.96 (0.90 to 1.02)	0.145
Smoking				
Previous	1.31 (0.79 to 2.19)	0.299	1.20 (0.84 to 1.71)	0.313
Current	1.03 (0.12 to 8.99)	0.980	1.12 (0.27 to 4.59)	0.876
APOE ε4 carrier	1.38 (0.76 to 2.53)	0.293	1.51 (1.00 to 2.27)	0.050
Diabetes	1.26 (0.51 to 3.09)	0.616	1.23 (0.65 to 2.33)	0.516
Heart failure	1.25 (0.53 to 2.95)	0.614	1.50 (0.83 to 2.70)	0.181
Hypertension	0.79 (0.47 to 1.32)	0.370	0.76 (0.53 to 1.09)	0.133
BMI	0.99 (0.94 to 1.04)	0.677	0.98 (0.94 to 1.02)	0.242
Depression	1.48 (0.76 to 2.88)	0.254	1.40 (0.88 to 2.25)	0.156
Social activity (hours per week)	0.74 (0.47 to 1.16)	0.191	1.00 (0.75 to 1.33)	0.987
Cognitive activity (hours per week)	0.89 (0.59 to 1.33)	0.579	1.00 (0.75 to 1.33)	0.996
Physical activity (hours per week)	0.98 (0.90 to 1.06)	0.632	0.98 (0.93 to 1.04)	0.551

Reference Class 1 (Stable: episodic memory *n*= 708; odor identification *n*= 646.
Abbreviations: *APOE* ε4, apolipoprotein epsilon 4; BMI, body mass index.
[a]N= 109 missing information on any 1 or more predictors.

Supplementary Table 4. Parameter estimates for joint trajectories in episodic memory and odor identification by latent class.

Parameters	Functions	Class 1- joint stable *n*=731 (71.5%) mean (SE)	Class 2 - OI decline *n*=203 (19.8%) mean (SE)	Class 3- joint decline *n*= 89 (8.7%) mean (SE)
**Fixed effects**				
Intercept	Episodic memory	0.430	-0.015	-0.107
	Odor identification	0.353	-0.777	-0.823
Linear annual rate of decline	Episodic memory	0.065	0.012	-0.148
	Odor identification	0.032	-0.137	-0.006
Quadratic annual rate of decline	Episodic memory	-0.010	0.012	-0.034
	Odor identification	-0.009	-0.006	-0.044
**Random effects**				
Intercept variance	Episodic memory	0.125	0.346	0.312
	Odor identification	0.097	0.801	1.103
Linear slope variance	Episodic memory	0.004	0.004	0.004
	Odor identification	0.004	0.004	0.004
Residual variance baseline	Episodic memory	0.077	0.079	0.077
	Odor identification	0.391	0.384	0.391
Residual variance follow up 1	Episodic memory	0.086	0.09	0.086
	Odor identification	0.263	0.26	0.263
Residual variance follow up 2	Episodic memory	0.089	0.09	0.089
	Odor identification	0.324	0.33	0.324
Residual variance follow up 3	Episodic memory	0.086	0.09	0.086
	Odor identification	0.380	0.38	0.380
Residual variance follow up 4	Episodic memory	0.090	0.09	0.090
	Odor identification	0.410	0.41	0.410
Residual variance follow up 5	Episodic memory	0.078	0.08	0.078
	Odor identification	0.348	0.35	0.348
Residual variance follow up 6	Episodic memory	0.099	0.10	0.099
	Odor identification	0.439	0.45	0.439
Residual variance at follow up 7	Episodic memory	0.108	0.10	0.108
	Odor identification	0.424	0.39	0.424

[a]*n* was based on the final class counts of the estimated model. Note that individuals are in fact assigned a probability of class membership.
